# Chronic NKG2D Engagement *In Vivo* Differentially Impacts NK Cell Responsiveness by Activating NK Receptors

**DOI:** 10.3389/fimmu.2017.01466

**Published:** 2017-11-03

**Authors:** Christine Koch, Younghoon Kim, Tobias Zöller, Christina Born, Alexander Steinle

**Affiliations:** ^1^Institute for Molecular Medicine, Goethe-University Frankfurt am Main, Frankfurt am Main, Germany; ^2^Department of Internal Medicine I, Division of Gastroenterology and Hepatology, University Hospital Frankfurt am Main, Frankfurt am Main, Germany

**Keywords:** NKG2D, NK cells, MICA, signal transduction, NK receptors

## Abstract

Immunosuppression is a typical hallmark of cancer and frequently includes perturbations of the NKG2D tumor recognition system as well as impaired signaling by other activating NK cell receptors. Several *in vitro* studies suggested that sustained engagement of the NKG2D receptor, as it is occurring in the tumor microenvironment, not only impairs expression and function of NKG2D but also impacts signaling by other activating NK receptors. Here, we made use of a transgenic mouse model of ubiquitous NKG2D ligand expression (H2-K^b^-MICA mice) to investigate consequences of chronic NKG2D engagement *in vivo* for functional responsiveness by other activating NK receptors such as NKp46 and Ly49D. Unexpectedly, we found no evidence for an impairment of NKp46 expression and function in H2-K^b^-MICA mice, as anticipated from previous *in vitro* experiments. However, we observed a marked downregulation and dysfunction of the activating receptor Ly49D in activated NK cells from H2-K^b^-MICA mice. Ly49D shares the adaptor proteins DAP10 and DAP12 with NKG2D possibly explaining the collateral impairment of Ly49D function in situations of chronic NKG2D engagement. Altogether, our results demonstrate that persistent engagement of NKG2D *in vivo*, as often observed in tumors, can selectively impair functions of unrelated NK receptors and thereby compromise NK responsiveness to third-party antigens.

## Introduction

Severe immunosuppression frequently occurs in patients with cancer (1, 2). However, our knowledge of the underlying mechanisms is still incomplete. NK cells with their ability to recognize and kill malignant cells by the mechanisms of “missing-self” recognition and “induced-self” recognition play an important role in the immunosurveillance of cancer (3–6). NK cell activation is controlled by a plethora of activating and inhibitory receptors (7, 8). Inhibitory NK cell receptors specific for MHC class I molecules mediate “missing-self” recognition by unleashing NK cell cytotoxicity against tumor cells having lost MHC class I surface expression (9). In parallel, such an NK reactivity toward malignant cells must be triggered by activating NK cell receptors ligating cell surface molecules on tumor cells. Preferential NK cell recognition of tumor cells is achieved by activating NK cell receptors binding to molecules induced or upregulated in the course of malignant transformation (“induced-self” recognition) (5, 6, 10).

The paradigmatic NK receptor for induced-self recognition is NKG2D, an activating receptor expressed on virtually all NK cells, but also on most other cytotoxic lymphocytes. In humans, NKG2D is expressed by nearly all NK cells, CD8 T cells, and γδ T cells, while in mice, NKG2D is not expressed by naïve CD8 T cells, unless they become activated (11, 12). In humans, NKG2D solely signals *via* the associated adaptor protein DAP10 (13), while NKG2D on activated mouse NK cells additionally employs the ITAM-bearing DAP12 adaptor for signaling (14, 15). NKG2D binds to several MHC class I-related cell surface glycoproteins, distinct in humans and in mice, which are not or barely expressed on “healthy” cells (10, 11, 16, 17). Yet, upon cellular stress, viral infection, or malignant transformation, the expression of NKG2D ligands (NKG2DL) is strongly induced and their upregulation at the cell surface efficiently promotes cytolysis of such “harmful” cells through engagement of NKG2D on cytotoxic lymphocytes (5, 16, 18, 19). NKG2D-deficient mice have provided evidence that NKG2D is involved in the immunosurveillance of tumor cells (20, 21). Certain viruses and many tumors counteract NKG2D-mediated elimination by various mechanisms, such as intracellular retention or proteolytic shedding of NKG2DL (10, 22–25). In addition, several *in vitro* and *in vivo* studies have shown that sustained engagement of NKG2D by membrane-bound NKG2DL, as it occurs in the tumor microenvironment, leads to silencing of NKG2D-mediated responses presumably by chronic receptor internalization and degradation (26–31). In addition, some of these *in vitro* studies have shown that sustained NKG2D engagement by NKG2DL not only impairs NKG2D function but also NK responsiveness mediated by other activating NK cell receptors, presumably by interfering with the expression of the respective signaling adaptors (27, 28, 32). One of these studies provided evidence that chronic NKG2D engagement promotes CD3ζ degradation in human NK cells and thereby paralyzes NK cell activation *via* CD3ζ-associated NK receptors such as NKp46 and NKp30 (32). Of note, CD3ζ chain downregulation has been described for various types of cancer and autoimmune diseases (33–35). CD3ζ is a signaling adaptor that is an essential part of TCR/CD3 complex, where it forms homodimers or heterodimers with CD3ε (36). In addition, CD3ζ is also expressed by NK cells where it acts as a signal transducer for some activating receptors such as NKp46 (7, 37). The mechanisms by which CD3ζ is downregulated in cancer patients are yet unclear. However, it is suggestive that loss of CD3ζ in tumor-infiltrating lymphocytes severely impairs anti-tumor immunity by T cells and NK cells (35). Building on the *in vitro* studies showing “cross-silencing” of other NK receptors as consequence of persistent NKG2D engagement, we wondered whether this mechanism may partially account for the observed CD3ζ degradation and concomitant functional impairments of T and NK cells in tumors of cancer patients.

To test this hypothesis, we employed a transgenic mouse model previously characterized in our laboratory where the human NKG2DL MICA is constitutively and ubiquitously expressed under control of the MHC class I promoter H2-K^b^ (31, 38). MICA (MHC class I chain-related protein A) is the best studied human NKG2DL, and frequent MICA expression by tumor cells and in cancer patients has been documented by many reports (39–41). Sustained engagement of NKG2D by MICA was shown to cause receptor internalization and degradation *in vitro* (22). In H2-K^b^-MICA mice, the constitutive expression of MICA*07 which functionally interacts with mouse NKG2D results in systemic NKG2D downregulation and dysfunction (31, 38). Nevertheless, these mice show a normal phenotype and no overt signs of autoimmunity, impaired immune function, or spontaneous carcinogenesis (31, 38). Using this mouse model, we addressed consequences of chronic NKG2D engagement for functional responsiveness of various activating receptors on mouse NK cells. Primarily, we focused on NKp46 expression and function, as NKp46 is the only known activating NK receptor in mice assembling with and signaling through CD3ζ (7, 37, 42). In addition, we addressed expression and functionality of the activating receptors NK1.1 and Ly49D to cover also other signaling pathways used by NK cells. While NK1.1 pairs and signals through FcRγ (43), Ly49D, like mouse NKG2D, assembles with and signals *via* both DAP10 and DAP12 (14, 15, 44).

## Materials and Methods

### Cells

All cell culture media were supplemented with 10% fetal calf serum (FCS) (Biochrom, Berlin, Germany), 2 mM glutamine, 100 U/ml penicillin, 100 µg/ml streptomycin (Sigma-Aldrich, Steinheim, Germany), and 1 mM sodium pyruvate (Life Technologies, Darmstadt, Germany). P815 is an FcγR^+^ murine mastocytoma cell line (ATCC TIB-64) and was maintained in RPMI 1640 (Sigma-Aldrich). Mock- or MICA*01-transduced B16F10 cells were kindly provided by Dr. Mathieu Blery, Innate Pharma, Marseille, and cultured in DMEM with non-essential amino acids (both from Sigma-Aldrich).

### Animals

Transgenic H2-K^b^-MICA mice expressing the human MICA*07 cDNA under control of the H2-K^b^ promoter have been described elsewhere (31). H2–K^b^–MICA mice have been continuously backcrossed with C57BL/6 mice since 1998. Litters were tested for MICA transgenes by PCR using genomic tail DNA or for MICA surface expression on PBL by flow cytometry using anti-MICA mAb AMO1-AlexaFluor647. Animals were maintained under specific pathogen-free conditions in the animal facilities of the University Hospital Frankfurt am Main, Germany. Animal experiments were approved by the local authorities (Regierungspräsidium Darmstadt, Germany, permit nos. F146/Anz.04, FU/Anz.1035 and FU/1115) and performed in full compliance with the respective national guidelines.

### NK Cells

Splenocytes were prepared by passing the spleen through a 100-µm nylon mesh and subsequent washing with ice-cold phosphate-buffered saline (PBS). Erythrocytes were removed from splenocytes by gradient centrifugation with Ficoll-Paque Plus (GE Healthcare Europe, Munich, Germany). NK cells were enriched from purified splenocytes using the mouse NK cell isolation kit II (Miltenyi Biotech, Bergisch Gladbach, Germany) according to the manufacturer’s protocol. The purity of isolated CD3^−^NK1.1^+^ NK cells was monitored by flow cytometry and varied between 60 and 85%.

### Antibodies

Anti-CD3ε-PerCP (145-2C11), anti-IFNγ-FITC (XMG1.2), anti-NKp46 (29A1.4), anti-NKG2D-PE (C7), anti-Ly49D (4E5), anti-Ly49D-PE (4E5), anti-Ly49H-PE (3D10), anti-NK1.1 (PK136), anti-CD49b-PE (DX5), anti-CD49b-FITC (DX5), anti-CD3ζ (6B10.2), anti-CD16/32-PE (93), and anti-CD16.2 (9E9) were purchased from BioLegend (San Diego, CA, USA). Anti-NKG2D (A10), anti-NK1.1-APC (PK136), anti-CD3ζ-PE (6B10.2), and Fixable Viability Dye eFluor 506 were obtained from eBioscience (San Diego, CA, USA). Anti-CD107a-APC was purchased from SouthernBiotech (Birmingham, AL, USA) and anti-NKp46-V450 (29A1.4) from BD Biosciences (San Jose, CA, USA). The MICA-specific mAb AMO1 was previously described (24) and conjugated with AlexaFluor647 according to standard protocols.

### Flow Cytometry

Cells were washed with ice-cold fluorescence-activated cell sorter (FACS) buffer (PBS, 2% FCS, 2 mM EDTA, 0.01% sodium azide). Prior to staining of surface receptors, cells were incubated with anti-CD16.2 (9E9) to block unspecific binding. Cells were then stained with relevant antibodies for 30 min at 4°C and washed again with FACS buffer. For intracellular staining, cells were fixed and permeabilized with Cytofix/Cytoperm (BD Biosciences) for 20 min on ice. Cells were incubated with antibodies for 45 min at 4°C and washed with saponin buffer (PBS, 0.5% bovine serum albumin, 0.1% saponin, 0.01% sodium azide). For intracellular staining of CD3ζ, splenocytes were preactivated *in vivo* by intraperitoneal (i.p.) injection of 5 µg/g body weight poly(I:C) (Sigma-Aldrich) 16 h before analysis. Otherwise, NK cells were activated *in vivo* by i.p. injection of 10 µg/g body weight poly(I:C) (Invivogen, Toulouse, France) 16 h before analysis. Cells were stained with DAPI or Fixable Viability Dye eFluor 506 (eBioscience) to assess viability. Flow cytometry analysis was performed with a FACS Canto II (BD Biosciences) and data analyzed using FlowJo (Tree Star, Ashland, OH, USA). The specific fluorescence intensity (SFI) was calculated by subtracting the mean fluorescence intensity (MFI) of the isotype control from the MFI of the antibody of interest.

### Redirected Cytolysis

Freshly isolated and purified mouse NK cells were tested for their capacity to trigger NK cytotoxicity in redirected cytolysis assays with antibody-loaded FcγR^+^ P815 cells as target cells. NK cells were preactivated *in vivo* by poly(I:C) injection 16 h before harvest. P815 cells were labeled with 50 μCi of ^51^Cr (Perkin Elmer, Waltham, MA, USA) for 2 h at 37°C. After washing, ^51^Cr-labeled P815 cells (targets) were co–cultured for 4 h with purified NK cells (effectors) in the presence of 1 µg/ml of the indicated antibodies at different effector (E) to target (T) ratios. Subsequently, supernatants were mixed with OptiPhase Supermix scintillation mixture in an IsoPlate-96 and measured with a MicroBeta2 plate counter (all from PerkinElmer). Spontaneous chromium release of target cells was always less than 15% of the maximum release of target cells lysed in 1% Triton X-100. Percentage of lysis was calculated as follows: 100 × (experimental release − spontaneous release)/(maximum release − spontaneous release). Experiments were performed in triplicates.

### Degranulation and IFNγ Secretion of NK Cells

MaxiSorb 96 well flat bottom plates (Nunc, Thermo Fisher Scientific, Waltham, MA, USA) were coated with 10 µg/ml PBS of the indicated antibodies for 16 h at 4°C and subsequently blocked by incubation with 10% FCS for 20 min. Splenocytes isolated from mice were added to the plates and incubated with 2 µg/ml anti-CD107a APC antibody and GolgiStop (BD Biosciences) for 4 h at 37°C. Stimulation with 25 ng/ml PMA plus 500 ng/ml ionomycin (both Sigma-Aldrich) served as positive control. Subsequently, splenocytes were stained with both anti-CD3-PerCP and anti-CD49b-PE, permeabilized, and stained with anti-IFNγ-FITC as described for the intracellular staining.

### Immunoblotting

For immunoblot analysis, purified NK cells from spleens of naïve mice were lysed using Pierce RIPA buffer (Thermo Fisher Scientific), containing the Complete protease inhibitor cocktail (Roche, Mannheim, Germany). 20 µg of total lysates were separated *via* non-reducing SDS-PAGE and transferred to PVDF membranes (Carl Roth, Arlesheim, Switzerland) by semi-dry blotting. Membranes were probed with 0.5 µg/ml anti-CD3ζ (6B10.2) at 4°C for 16 h, followed by detection with horseradish peroxidase-conjugated goat anti-mouse IgG antibody (Jackson ImmunoResearch Laboratories, West Grove, PA, USA). Immunoblot signals were generated with Femto-ECL© (Thermo Fisher Scientific).

### Tumor Inoculation

B16F10-MICA*01 or B16F10-mock cells were washed with PBS and resuspended in PBS at 1 × 10^5^ cells/ml. 100 µl B16F10 cells (1 × 10^4^) were mixed with 100 µl Matrigel (Corning, Corning, NY, USA) and the mixture injected subcutaneously into the flank of mice. Tumor growth was monitored daily and tumor size documented every second day by measuring tumor surface with a metric caliper. Tumor size [mm] was calculated according to the formula (width of tumor (W) [mm] + length of tumor (L) [mm])/2 = tumor size [mm]. Growth rates of tumors were determined as described (45) by calculating the slope of the curve of the log-transformed tumor volumes. According to the animal guidelines, mice were sacrificed when tumor size exceeded 14 mm.

### Statistics

Statistical analyses as detailed in figure legends were performed using Prism 7 (GraphPad, San Diego, CA, USA).

## Results

### Persistent NKG2D Engagement *In Vivo* Differentially Affects Surface Expression of Activating NK Receptors

**Figure 1:**
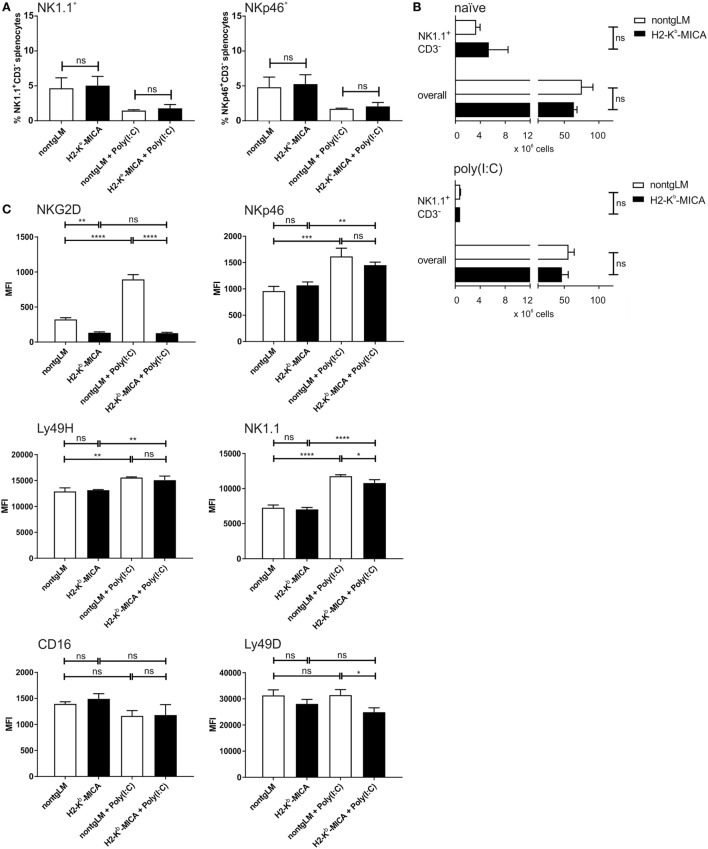
Reduced NK cell surface expression of NK1.1 and Ly49D in mice with enforced ligand-induced NKG2D downregulation. Numeric and phenotypic assessment of NK cells from H2-K^b^-MICA mice by flow cytometry. Splenic NK cells were isolated either from naïve mice or 16 h after poly(I:C) injection (activated NK cells). **(A,B)** No significant differences in frequencies **(A)** or total numbers **(B)** between resting or activated splenic NK cells from H2-K^b^-MICA mice and non-transgenic littermates (non-tgLM). NK cells were gated as NK1.1^+^CD3^−^ splenocytes, and in panel (A) also as NKp46^+^CD3^−^ splenocytes. Data show means plus SE from three mice analyzed in parallel. One representative out of at least three independent experiments is shown. A two-way ANOVA was performed with a Sidak posttest. **(C)** Surface expression of activating receptors NKG2D, NKp46, Ly49D, Ly49H, NK1.1, and CD16 on splenic NK cells from naïve or poly(I:C) injected H2-K^b^-MICA mice versus non-tgLM. Data show means plus SDs from three mice analyzed in parallel. One representative out of at least three independent experiments is shown. Two-way ANOVA was performed with a Tukey posttest to assess statistical significance of differences (ns = not significant).

To investigate the impact of chronic NKG2D engagement on the NK cell compartment *in vivo*, we studied abundance, phenotype, and function of splenic NK cells from H2-K^b^-MICA which are NKG2D-dysfunctional and exhibit impaired NK and memory T cell responses to NKG2DL^+^ tumors (31, 38). At first, abundance of splenic NK cells in H2-K^b^-MICA mice was compared to nontransgenic littermates (non-tgLM). No significant differences were observed in frequencies of NK cells among splenocytes when gating on either NK1.1^+^CD3^−^ or NKp46^+^CD3^−^ splenocytes, respectively (Figure 1A). In addition, absolute numbers of neither NK1.1^+^CD3^−^ splenic NK cells nor total splenocytes varied significantly between MICA-transgenic mice and non-tgLM mice (Figure 1B). This was also true for splenocytes isolated 16 h after poly(I:C) injection (Figure 1B). However, as previously described (31), NKG2D receptors were profoundly downregulated on H2-K^b^-MICA NK cells as a direct consequence of persistent engagement by ubiquitously expressed MICA*07 (Figure 1C). Previous *in vitro* studies where mouse NK cells were co-cultured with NKG2DL-overexpressing cells (i.e., RMA-H60a) showed that ligand-induced NKG2D downregulation not only impairs surface expression and signaling of NKG2D receptors, but also impacts function of other activating NK receptors by negatively affecting expression of their adaptor chains DAP10, DAP12, and CD3ζ, respectively (28), a phenomenon that has been termed “cross-tolerance” (27). In order to assess whether such a cross-tolerance upon persistent NKG2D engagement also occurs *in vivo*, we first determined surface expression levels of activating NK receptors NKp46, CD16, Ly49D, and Ly49H, respectively, on splenic NK cells from H2-K^b^-MICA mice and compared these to levels on NK cells from non-transgenic littermates (non-tgLM).

No significant alterations in the cell surface expression levels of any of these activating receptors on splenic NK cells from H2-K^b^-MICA were observed (Figure 1C). We reasoned, however, that enforced NKG2DL-induced downregulation of NKG2D may be required *in vivo* in order to induce significant effects of NKG2D-mediated cross-tolerance. Hence, we sought to enforce NKG2DL-induced NKG2D downregulation in H2-K^b^-MICA mice by enhancing NKG2D expression. We had previously observed that *in vivo* activation of NK cells upon poly(I:C) injection results in a strong NKG2D upregulation in non-transgenic mice, while residual NKG2D surface expression by H2-K^b^-MICA NK cells remained unaltered (31). This is most likely due to the persistent and strong engagement of transgenic MICA that shows increased expression upon poly(I:C) injection (data not shown), resulting in an enhanced downregulation and internalization of NKG2D (31).

Therefore, we injected H2-K^b^-MICA mice and non-tgLM with poly(I:C) and, after 16 h, determined surface expression levels of activating NK receptors. As expected, activated NK cells from non-tgLM showed a marked NKG2D upregulation, while NKG2D expression on H2-K^b^-MICA NK cells remained unaltered. Hence, the difference in NKG2D levels between H2-K^b^-MICA NK cells and non-tgLM increased from twofold on naïve NK cells to sevenfold on poly(I:C)-activated NK cells (Figure 1C).

Poly(I:C) injection also resulted in an upregulated surface expression of NKp46, NK1.1, and Ly49H on activated splenic NK cells of both non-tgLM and H2-K^b^-MICA mice. By contrast, Ly49D surface expression was not enhanced upon poly(I:C) injection and CD16 surface expression appeared even reduced, even though this trend did not reach statistical significance (Figure 1C). However, most importantly, Ly49D expression levels on activated H2-K^b^-MICA NK cells were markedly lower than on naïve or activated non-tgLM NK cells, indicating that Ly49D surface expression is affected by NKG2DL-induced NKG2D downregulation *in vivo*. A significantly lower surface expression was also observed for NK1.1 on activated H2-K^b^-MICA NK cells as compared to activated NK cells from non-tgLM (Figure 1C).

Collectively, persistent ligand engagement of NKG2D *in vivo* did not significantly affect surface expression of NKp46, Ly49H, and CD16, while expression levels of activating receptors Ly49D and NK1.1 were markedly reduced when NKG2D engagement on H2-K^b^-MICA NK cells was enforced through poly(I:C)-induced NKG2D upregulation.

### Chronic Engagement of NKG2D *In Vivo* Does Not Affect NKp46-Mediated Cytotoxicity

A previous *in vitro* study has linked chronic stimulation of NKG2D on human NK cells to a functional impairment of the NK receptors CD16, NKp30, and NKp46 (32). The authors had provided evidence that chronic NKG2D stimulation causes the enhanced degradation of the CD3ζ adaptor protein, thereby reducing the responsiveness of the CD3ζ-associated receptors CD16, NKp30, and NKp46, respectively. Hence, we wondered whether such a “cross-silencing” of CD3ζ-associated NK receptors also occurs in our *in vivo* model of chronic NKG2D engagement. Since NKp30 is not expressed in *mus musculus* (46) and CD16 pairs in mice only with FcRγ, but not with CD3ζ (37), we focused on functional responsiveness of NKp46.

**Figure 2:**
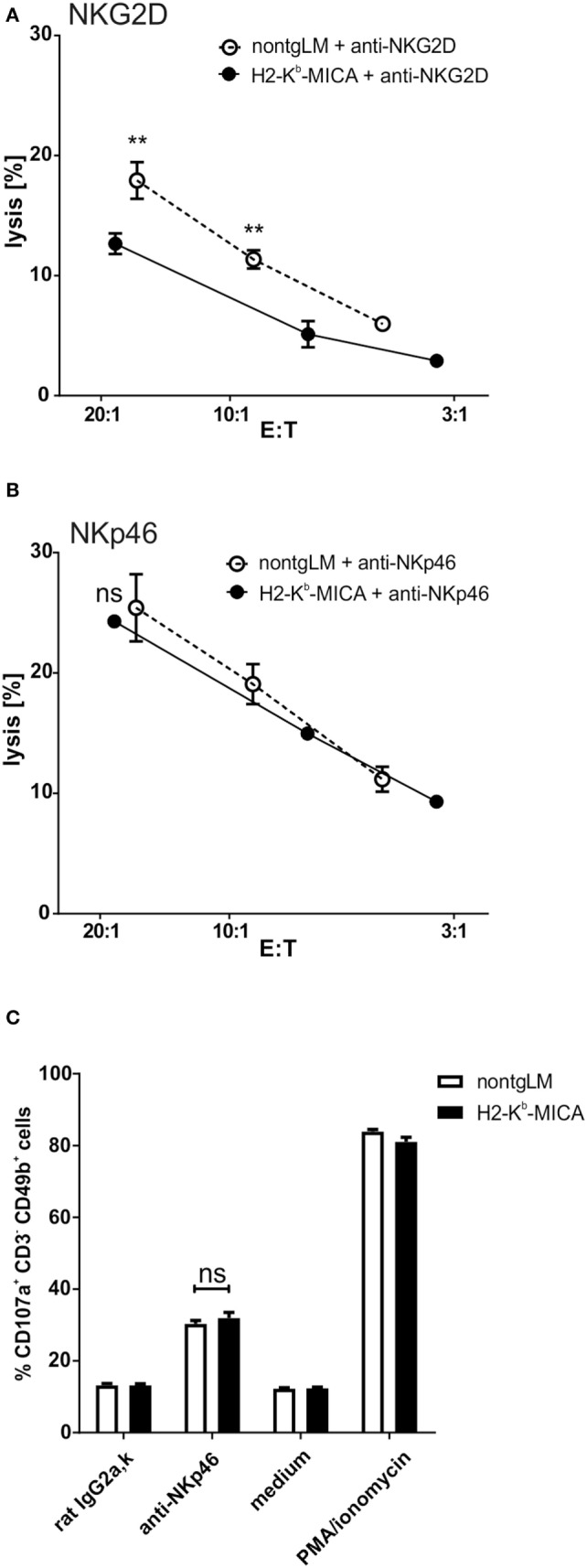
Chronic engagement of NKG2D *in vivo* does not affect NKp46-mediated cytotoxicity. **(A,B)** Redirected cytolysis assay with antibody-loaded P815 cells and *in vivo* preactivated splenic NK cells [poly(I:C) injection 16 h prior to isolation] from H2-K^b^-MICA mice and non-transgenic littermates (non-tgLM), respectively. Data represent means with SDs. One representative out of at least four independent experiments is shown. Two-way ANOVA with a Sidak posttest was applied. **(C)** Degranulation (CD107a surfacing) of *in vivo* preactivated splenic NK cells after 4 h stimulation with plate-bound anti-NKp46 antibodies. Medium or stimulation with PMA/ionomycin served as controls. **(A–C)** Data represent means of triplicates with SDs. One representative out of at least three independent experiments is shown. Statistical significance was assessed with two-way ANOVA with Sidak posttest.

To this aim, we addressed NKp46-mediated cytotoxicity by performing a redirected cytolysis assay. As previously reported, the cytotoxic response of NK cells from H2-K^b^-MICA was markedly reduced when NKG2D was cross-linked in a redirected lysis assay (Figure 2A). By contrast, cross-linking of NKp46 stimulated cytotoxicity of non-tg NK cells and H2-K^b^-MICA NK cells to a similar extent (Figure 2B). We sought to corroborate these results by assessing degranulation by flow cytometric analysis of CD107a surfacing. In accordance with the results from the cellular cytotoxicity assays, degranulation was comparable between H2-K^b^-MICA mice and non-transgenic controls when activated *via* NKp46 (Figure 2C).

### Chronic Engagement of NKG2D *In Vivo* Does Not Substantially Alter CD3ζ Expression in Splenic H2-K^b^-MICA NK Cells

**Figure 3:**
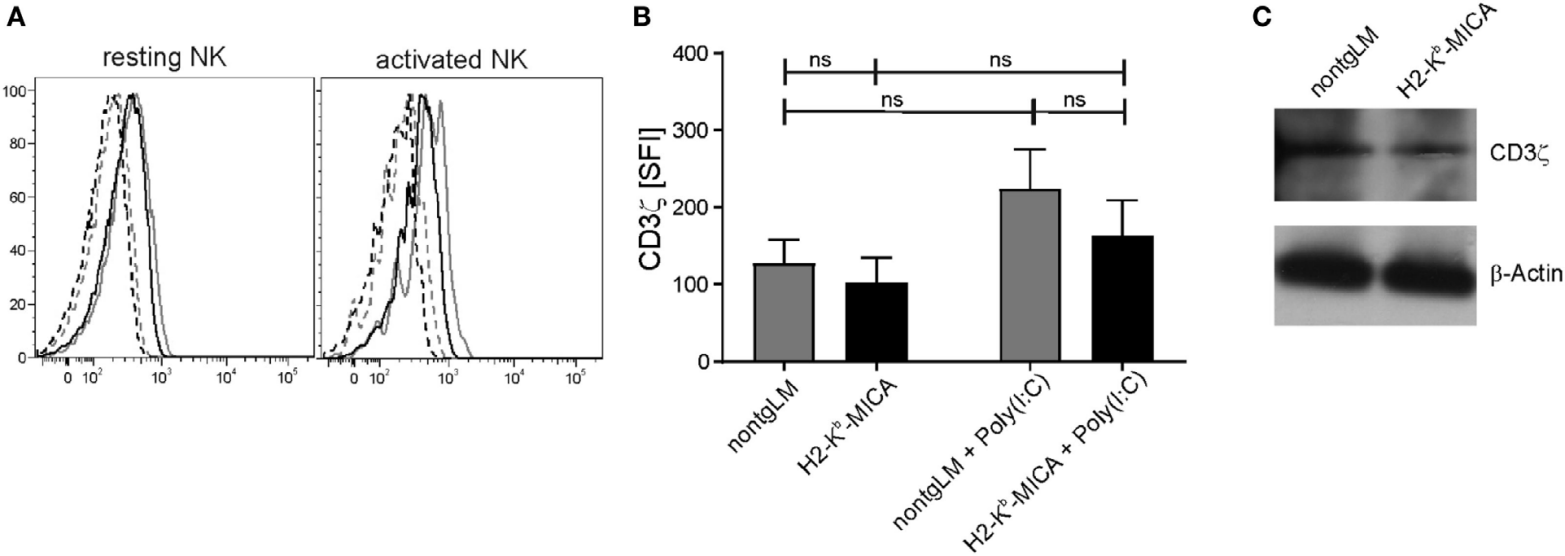
CD3ζ expression in splenic H2-K^b^-MICA NK cells is not substantially altered. **(A,B)** Splenic NK cells from H2–K^b^–MICA mice and non-transgenic littermates (non-tgLM), respectively, were analyzed for CD3ζ expression. Resting NK cells were from naïve mice, activated NK cells from mice injected with poly(I:C) 16 h prior to analysis. Splenocytes were permeabilized and stained for CD3ζ (mAb 6B10.2) by flow cytometry. **(A)** Representative CD3ζ stainings of resting (left) or activated (right) NKp46^+^ splenocytes from a H2–K^b^–MICA mouse (black solid line) and from a non-tgLM (gray solid line) overlayed with the corresponding isotype controls (stippled lines). **(B)** Summarized data from three mice per group analyzed in parallel showing means of specific fluorescence intensities (SFIs) plus SDs. Two-way ANOVA was performed with a Tukey posttest to assess statistical significance (ns = not significant). **(C)** Immunoblot analysis of lysates of freshly purified splenic NK cells from naïve H2-K^b^-MICA and non-tgLM mice with anti-CD3ζ mAb 6B10.2. Purified NK cells from three mice per group were pooled for analysis. Actin was probed for control. One representative of three experiments is shown.

As we had neither observed an impact of chronic *in vivo* NKG2D engagement on NKp46 surface expression nor on NKp46-mediated cytotoxicity, we wondered whether CD3ζ expression levels may be affected by chronic NKG2D stimulation in H2-K^b^-MICA mice as described by Hanaoka and colleagues in the human setting (32). To this aim, we analyzed expression of CD3ζ in H2-K^b^-MICA NK cells by intracellular staining and immunoblotting. CD3ζ levels appeared slightly lower in resting and activated NK cells of H2-K^b^-MICA mice as compared to non-tg NK cells; however, differences were not statistically significant (Figures 3A,B). In agreement with the results gained by flow cytometry, immunoblotting of NK lysates of purified splenic NK cells did not reveal substantial differences in CD3ζ expression levels of H2-K^b^-MICA NK cells versus non-tg NK cells (Figure 3C).

### Chronic Engagement of NKG2D *In Vivo* Does Not Affect Tumor Growth of B16 Melanoma Cells

**Figure 4:**
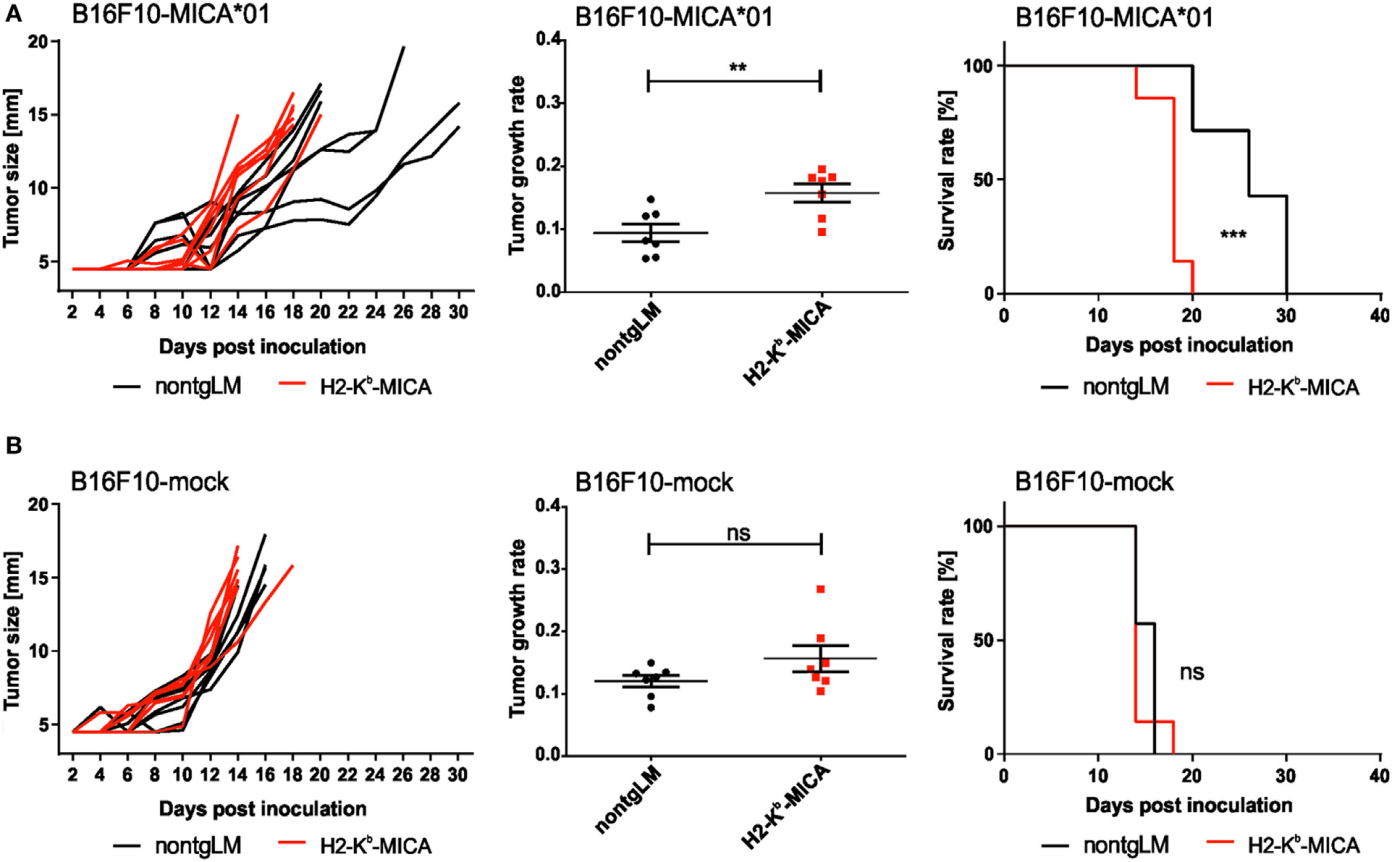
Chronic engagement of NKG2D *in vivo* does not affect tumor growth of B16F10 melanoma. **(A,B)** H2-K^b^-MICA mice (red lines/dots) and non-transgenic littermates (non-tgLM) (black lines/dots) were inoculated subcutaneously with **(A)** 1 × 10^4^ B16F10-MICA*01 or **(B)** 1 × 10^4^ B16F10-mock cells. Tumor size was measured every second day using a metric caliper and mice with a tumor size exceeding 14 mm were euthanized. Tumor growth (left panels), tumor growth rate (middle), and survival (right) is shown for seven mice per group. Growth rates of tumors were calculated as described in “Materials and Methods” section. Statistical significance of differences in tumor growth rate or in survival was calculated using unpaired *t*-test or log-rank (Mantel–Cox) test, respectively.

Finally, we sought to address a functional “cross-tolerance” of CD3ζ-associated NKp46 in H2-K^b^-MICA mice in a tumor rejection experiment. As H2-K^b^-MICA mice are impaired in rejection of tumors expressing NKG2D ligands (NKG2DL), we selected the melanoma cell line B16F10 that is reportedly devoid of NKG2DL, but expresses yet uncharacterized ligands for NKp46 (47, 48). In accordance with previous observations (31), growth of B16F10 tumor cells ectopically expressing the human NKG2DL MICA*01 was accelerated in NKG2D-dysfunctional H2-K^b^-MICA mice as compared to non-tgLM (Figure 4A). However, there was no significant difference in tumor growth of mock-transduced B16F10 cells between H2-K^b^-MICA mice and control mice, thus not providing *in vivo* evidence for a “cross-tolerance” of NKp46 through chronic NKG2D stimulation (Figure 4B).

### Chronic Engagement of NKG2D *In Vivo* Impairs Ly49D-Mediated Cytotoxicity

**Figure 5:**
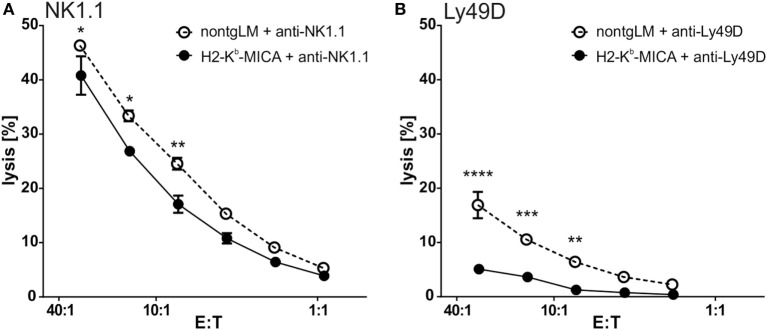
Ly49D-mediated cytotoxicity is severely impaired by chronic engagement of NKG2D *in vivo*. **(A,B)** Redirected lysis of P815 cells loaded with **(A)** anti-NK1.1 or **(B)** anti-Ly49D antibodies by poly(I:C)-activated splenic NK cells. Data represent means of triplicates with SDs. One representative out of four independent experiments is shown. Two-way ANOVA was performed with a Sidak posttest for comparison.

As we could not observe any effects of NKG2DL-induced NKG2D downregulation on NKp46 expression or function, we moved on to investigate the potential interference of chronic NKG2D engagement with other activating NK receptor signaling pathways. Previous *in vitro* studies had suggested that chronic NKG2D engagement may affect both DAP10/DAP12-dependent and DAP10/DAP12-independent NK receptor signaling (27, 28). Hence, we addressed the responsiveness of the activating receptors NK1.1 or Ly49D in redirected lysis assays with NK cells from H2-K^b^-MICA mice. While NK1.1 signals *via* FcRγ, an adaptor protein not used by NKG2D, Ly49D signals *via* DAP12 and DAP10 and thus sharing usage of adaptor proteins with NKG2D. Of note, in redirected lysis assays addressing NK1.1 responsiveness, the lytic capacity of H2-K^b^-MICA NK cells was slightly reduced in comparison to the cytotoxic response of control NK cells (Figure 5A). However, when assessing Ly49D responsiveness, Ly49D ligation of NK cells from H2-K^b^-MICA mice did not give rise to a marked redirected lysis, in contrast to non-tg NK cells (Figure 5B), showing potent “cross tolerization” of Ly49D signaling in situations of chronic NKG2D engagement.

### Chronic Engagement of NKG2D *In Vivo* Affects IFNγ Secretion of NK Cells

**Figure 6:**
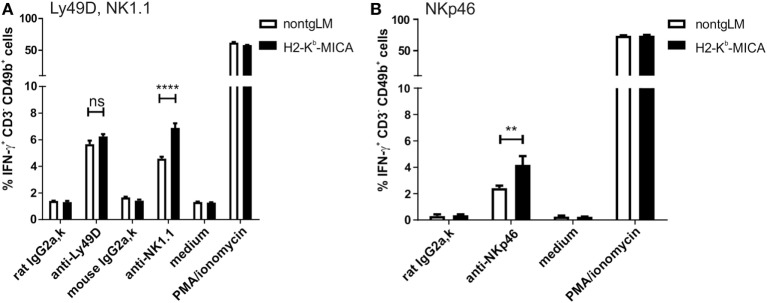
Increased frequencies of IFNγ-producing NK cells in H2-K^b^-MICA mice upon triggering NK1.1 and NKp46. **(A,B)** Freshly isolated splenic NK cells from H2-K^b^-MICA mice or non-transgenic littermates (non-tgLM) were stimulated for 4 h with plate-bound **(A)** anti-Ly49D or anti-NK1.1 or **(B)** anti-NKp46, subsequently frequencies of IFNγ-producing NK cells (CD3^−^CD49b^+^) determined by flow cytometric analysis of permeabilized cells. Stimulations with isotype controls (IgG2a), medium, or PMA/ionomycin were included as controls. Data represent means of triplicates with SDs. *P* values were determined using two-way ANOVA with a Sidak posttest for multiple comparisons.

A major effector mechanism of NK cells apart from cellular cytotoxicity is the secretion of cytokines such as IFNγ, TNF, and GM-CSF. While both cytotoxicity and cytokine secretion can be triggered by engagement of activating NK receptors, the corresponding signaling pathways and ensuing cellular responses are at least in part disparate. Hence, we wondered whether cytotoxicity and cytokine secretion of NK cells chronically stimulated *via* NKG2D *in vivo* is affected similarly. To this aim, we addressed secretion of IFNγ by splenic H2-K^b^-MICA NK cells preactivated by poly(I:C) injection *in vivo*. Unexpectedly, IFNγ responses of H2-K^b^-MICA NK cells upon Ly49D cross-linking were not significantly different from that of NK cells from control mice (Figure 6A). There were even stronger IFNγ responses of H2-K^b^-MICA NK cells upon cross-linking of either NK1.1 (Figure 6A) or NKp46 (Figure 6B) as compared to NK cells from non-tgLM indicating that chronic NKG2D engagement *in vivo* results in a higher frequency of NK cells responding to NK1.1 or NKp46 cross-linking with IFNγ secretion.

## Discussion

The potent activating immunoreceptor NKG2D specifically entails cytotoxic lymphocytes to react more robust against “dangerous” self-cells subjected to viral and bacterial infection, genotoxic stress, or other forms of cellular stress (5, 10). Since genotoxic stress often occurs in early stages of malignant transformations and has been shown to induce NKG2DL expression as does cellular proliferation, it has been proposed that NKG2D may be involved in early recognition and elimination of nascent tumors (5, 20). In fact, many established tumor cells *in situ* and tumor cell lines *in vitro* do express a heterogeneous set of NKG2DL and therefore are susceptible to NKG2D-mediated cytolysis (49, 50). However, it remains poorly understood why NKG2DL-expressing tumors are not eliminated by cytotoxic lymphocytes in cancer patients. Several tumor-mediated immune escape mechanisms targeting NKG2D-mediated immunosurveillance have been proposed based on *in vitro* studies (6, 16). However, most of these scenarios have not been assessed *in vivo*.

The importance of the *in vivo* testing of such hypothetical models of NKG2D immune escape was highlighted by the recent study from the Raulet laboratory where, contrary to current belief, release of a soluble NKG2DL by B16 tumor cells did not impair NK cell functions and tumor rejection, but rather resulted in an enhanced NK cell reactivity and faster tumor rejection (51). The authors do explain the beneficial effect of soluble NKG2DL release by the blockade of NKG2DL-induced engagement and downregulation of NKG2D in the tumor microenvironment. According to their hypothesis, the high affinity NKG2DL Mult-1 released by the tumor cells blocks the engagement of NKG2D on NK cells and T cells by the NKG2DL Rae-1 on tumor-resident host cells and thereby prevents the NKG2D-induced anergy of NK cells (51). Of note, NKG2D downregulation on peripheral blood lymphocytes is a common feature of cancer patients (22). However, it remains controversial whether NKG2D downregulation is consequence of soluble NKG2DL in the serum or due to NKG2DL engagement in the tumor bed.

Here, we address the issue, how NKG2DL-induced NKG2D downregulation *in vivo* affects the responsiveness of NK cells by other activating NK receptors using a transgenic mouse model of ubiquitous NKG2DL exposure. We observed that chronic NKG2D engagement by ubiquitously and persistently expressed MICA differentially affects the surface expression and function of activating mouse NK receptors. Unexpectedly, we did not observe an adverse effect on NKp46 expression and NKp46-mediated functional responsiveness of NK cells. On the other hand, we observed a pronounced reduction of Ly49D expression accompanied by an impairment of Ly49D-triggered NK cell cytotoxicity. We excluded a physical interaction between Ly49D and MICA that may have directly caused Ly49D downregulation (data not shown).

Since Ly49D and NKG2D share adaptor proteins DAP10 and DAP12 for surface expression and signaling, impaired Ly49D expression and function is likely due to enhanced degradation of DAP10 and/or DAP12 through chronic NKG2D engagement and internalization as shown *in vitro* (28, 29, 44). Ly49D surface expression and function was shown to depend more on DAP12 than on DAP10 (44). In case of Ly49H, the predominant association with DAP12 appears even more pronounced according to previous studies addressing surface expression of both Ly49D and Ly49H on DAP10- and/or DAP12-deficient mice (44) and co-immunoprecipitation of DAP10 and DAP12 with Ly49D and Ly49H (27), respectively. Hence, the differential impact of chronic NKG2D engagement on Ly49D versus Ly49H surface expression observed in our study may possibly be explained by a preferential degradation of DAP10 adaptor chains which are constitutively associated with NKG2D. By contrast, DAP12 can only be recruited by the short NKG2D-S form which is only generated by NK cells upon activation (14, 15). It remains unclear, why surface expression and function of NK1.1 is affected in H2-K^b^-MICA mice as NK1.1 is known to signal by the FcRγ chain and not *via* DAP10/DAP12. However, in support of these findings, Coudert and colleagues had reported NK1.1 downregulation as a consequence of chronic NKG2D engagement *in vitro* (27, 28). Extrapolating our findings to the human situation, one may predict that persistent engagement and downregulation of NKG2D in the tumor microenvironment may also affect the functionality of other activating receptors sharing signaling adaptors with NKG2D on cytotoxic lymphocytes, and, possibly, given the observed slight impairment of NK1.1 expression and function, even functionality of activating receptors using other adaptors such as FcRγ. However, as the NKG2D–NKG2DL system in humans differs in several aspects from the situation in mice, including a selective employment of DAP10 adaptors, the constitutive NKG2D expression on CD8 αβ T cells and non-homologous NKG2D ligands differing by expression pattern and NKG2D affinity from their mouse counterparts (10, 12, 16), consequences of persistent NKG2D engagement in human tumors may be substantially different to the mouse model and should be addressed by future studies. Unexpectedly, triggering of NKp46 and NK1.1, but not Ly49D, consistently led to an increased frequency of IFNγ-producing NK cells of H2-K^b^-MICA mice as compared to non-tg controls. Of note, Guerra and colleagues also described a higher frequency of IFNγ-producing NK cells from NKG2D-deficient mice upon cross-linking of NKp46, NK1.1, or Ly49D, respectively, and account it to an increased frequency of terminally mature CD27^lo^ NK cells in these mice (52). Hence, the observed higher rate of IFNγ-producing NK cells in H2-K^b^-MICA mice may rather be due to a slightly altered NK cell maturation associated with NKG2D dysfunction and not represent a consequence of disturbed signaling by the respective activating receptors.

In summary, in our *in vivo* model, exhibiting a constitutive NKG2DL expression and a consecutive persistent NKG2DL-induced NKG2D downregulation as it is observed in tumor patients, we could neither detect a functional impairment of the activating receptor NKp46 nor a marked CD3ζ downregulation even upon enforced chronic NKG2D engagement. However, NKG2D-mediated cross-tolerance *in vivo* could be shown for the activating receptor Ly49D sharing with NKG2D the requirement for the adaptor proteins DAP10 and DAP12.

## Ethics Statement

This study was carried out in accordance with current laws for animal research and experimental procedures approved by the Regierungspräsidium Darmstadt, Germany, permit nos. F146/Anz.04, FU/Anz.1035, and FU/1115.

## Author Contributions

CK, YK, TZ, and CB performed experiments and analyzed data; CK and AS designed research; CK, CB, and AS wrote the manuscript. All authors have read and approved the manuscript.

## Conflict of Interest Statement

The authors declare that the research was conducted in the absence of any commercial or financial relationships that could be construed as a potential conflict of interest.
